# Network Representations of Facial and Bodily Expressions: Evidence From Multivariate Connectivity Pattern Classification

**DOI:** 10.3389/fnins.2019.01111

**Published:** 2019-10-29

**Authors:** Yin Liang, Baolin Liu, Junzhong Ji, Xianglin Li

**Affiliations:** ^1^Faculty of Information Technology, Beijing Artificial Intelligence Institute, Beijing University of Technology, Beijing, China; ^2^Tianjin Key Laboratory of Cognitive Computing and Application, School of Computer Science and Technology, Tianjin University, Tianjin, China; ^3^School of Computer and Communication Engineering, University of Science and Technology Beijing, Beijing, China; ^4^State Key Laboratory of Intelligent Technology and Systems, National Laboratory for Information Science and Technology, Tsinghua University, Beijing, China; ^5^Medical Imaging Research Institute, Binzhou Medical University, Yantai, China

**Keywords:** facial expressions, bodily expressions, functional magnetic resonance imaging, functional connectivity, multivariate pattern classification

## Abstract

Emotions can be perceived from both facial and bodily expressions. Our previous study has found the successful decoding of facial expressions based on the functional connectivity (FC) patterns. However, the role of the FC patterns in the recognition of bodily expressions remained unclear, and no neuroimaging studies have adequately addressed the question of whether emotions perceiving from facial and bodily expressions are processed rely upon common or different neural networks. To address this, the present study collected functional magnetic resonance imaging (fMRI) data from a block design experiment with facial and bodily expression videos as stimuli (three emotions: anger, fear, and joy), and conducted multivariate pattern classification analysis based on the estimated FC patterns. We found that in addition to the facial expressions, bodily expressions could also be successfully decoded based on the large-scale FC patterns. The emotion classification accuracies for the facial expressions were higher than that for the bodily expressions. Further contributive FC analysis showed that emotion-discriminative networks were widely distributed in both hemispheres, containing regions that ranged from primary visual areas to higher-level cognitive areas. Moreover, for a particular emotion, discriminative FCs for facial and bodily expressions were distinct. Together, our findings highlight the key role of the FC patterns in the emotion processing, indicating how large-scale FC patterns reconfigure in processing of facial and bodily expressions, and suggest the distributed neural representation for the emotion recognition. Furthermore, our results also suggest that the human brain employs separate network representations for facial and bodily expressions of the same emotions. This study provides new evidence for the network representations for emotion perception and may further our understanding of the potential mechanisms underlying body language emotion recognition.

## Introduction

Humans can readily recognize others’ emotions and make the corresponding reactions. In daily communications, emotions can be perceived from facial and bodily expressions. The ability to decode emotions from different perceptual cues is a crucial skill for the human brain. In recent years, the representation mechanisms of facial and bodily expressions have been intensively explored, so as to deepen our understanding of the neural basis underlying this brain–behavior relationship.

Using functional magnetic resonance imaging (fMRI), neuroimaging studies have identified a number of brain regions showing preferential activation to facial and bodily expressions. An earlier model for face perception was proposed by Haxby et al. (2000) and Gobbini and Haxby (2007), which consisted of a “core” and an “extended” system. These face-selective areas, especially the occipital face area (OFA), the fusiform face area (FFA), and the posterior superior temporal sulcus (pSTS), which together constituted the core face network, have been considered as key regions in charge of processing the identity and emotional features of the face (Grill-Spector et al., 2004; Ishai et al., 2005; Lee et al., 2010; Gobbini et al., 2011). Bodies and body parts are found to be represented in the extrastriate body area (EBA) and the fusiform body area (FBA) (Downing and Peelen, 2016). Particularly, the FBA is partially overlapped with the FFA, and some similarities have been found between the processing of bodies and faces (Minnebusch and Daum, 2009; de Gelder et al., 2010). Because the fusiform gyrus (FG) contains both FFA and FBA, this area is considered to represent the characteristics of the whole person (Kim and McCarthy, 2016). Studies in macaques and humans have found that the STS, which acted as a crucial node for processing of social information, exhibited sensitivity to both faces and bodies (Pinsk et al., 2009). Recent studies have also proposed that the STS participated in the processing of facial and bodily motions, postures, and emotions (Candidi et al., 2011; Zhu et al., 2013).

As a data-driven technique, multivariate pattern analysis (MVPA) provides a promising method to infer the functional roles of the cortical areas and networks from the distributed patterns of the fMRI data (Mahmoudi et al., 2012). Recently, using MVPA, a growing number of studies have explored the emotion decoding based on the activation patterns. Said et al. (2010) and Harry et al. (2013) revealed the successful decoding of facial emotions in the STS and FFA, while Wegrzyn et al. (2015) directly compared the emotion classification rates across the face processing areas in Haxby’s model (Haxby et al., 2000). Our previous studies respectively, identified the face- and body-selective areas as well as the motion-sensitive regions and employed activation-based MVPA to explore their roles in decoding of facial and bodily expressions (Liang et al., 2017; Yang et al., 2018). However, these studies mainly focused on the emotion decoding from the activation patterns of specific brain regions. Due to the expected existence of interactions between different cortical regions, the potential contributions of the connectivity patterns in the processing of emotional information need to be further explored. In comparison with the studies on specific brain regions, functional connectivity (FC) analysis takes into account the functional interactions between distinct brain regions and thus can provide new insights into how large-scale neuronal communication and information integration relate to the human cognition and behavior. Commonly, FC can be effectively measured by the correlation analysis, which characterizes the temporal correlations in the fMRI activity between different cortical regions (Smith, 2012). In recent years, there has been increasing interest in FC analyses, and studies on the recognition of various objects have commonly observed intrinsic interconnections between distinct brain regions (He et al., 2013; Zhen et al., 2013; Hutchison et al., 2014). In addition to analyzing several predefined regions of interest (ROIs) or networks, whole-brain FC analysis can further ensure the optimal employment of the wealth of information present in the fMRI data (Zeng et al., 2012). Using whole-brain FC analysis combined with MVPA, recent fMRI studies have demonstrated the successful decoding of neurological disorders and various object categories from the FC patterns (Zeng et al., 2012; Liu et al., 2015; Wang et al., 2016). Inspired by these studies, our recent study has further revealed the successful decoding of facial expressions based on the FC patterns (Liang et al., 2018). However, the potential contribution of the FC patterns in the decoding of bodily expressions remains unclear, and no neuroimaging studies have resolved the question of whether emotions perceiving from facial and bodily expressions are processed rely upon common or different neural networks.

This study aimed to explore the network representations of facial and bodily expressions. To address this, we collected fMRI data in a block design multi-category emotion classification experiment wherein participants viewed emotions (anger, fear, and joy) from videos of facial and bodily expressions. Dynamic stimuli were employed in the present study as there was evidence that suggested that dynamic stimuli had greater ecological validity than their static counterparts and might be more appropriate to investigate the “authentic” mechanism of the brain (Johnston et al., 2013). We conducted whole-brain FC analysis to estimate the FC patterns for each emotion in each stimulus type, and employed multivariate connectivity pattern classification analyses (fcMVPA). We calculated the classification accuracies for facial and bodily expressions based on the FC patterns and constructed emotion-preferring networks by identifying the discriminative FCs.

## Materials and Methods

### Participants

Twenty-four healthy, right-handed subjects (12 females, range 19–25 years old) participated in this study. All subjects had normal or corrected-to-normal vision and with no history of neurological or psychiatric disorders. Informed consents were obtained from all individual participants included in the study. This experiment was conducted in accordance to the local Ethics Committee and was approved by the Research Ethics Committee of Yantai Affiliated Hospital of Binzhou Medical University. Four subjects were discarded due to the excessive head motion during the scanning (translation >2 mm, rotation >2°, Liu et al., 2015). Therefore, the final connectivity analysis included 20 subjects.

### fMRI Data Acquisition

Functional and structural images were acquired by a 3.0-T Siemens scanner with an eight-channel head coil in Yantai Affiliated Hospital of Binzhou Medical University. Foam pads and earplugs were used to reduce the head motion and scanner noise. Functional images were acquired using a gradient echo-planar imaging (EPI) sequence (TR = 2000 ms, TE = 30 ms, voxel size = 3.1 × 3.1 × 4.0 mm^3^, matrix size = 64 × 64, slices = 33, slice thickness = 4 mm, slice gap = 0.6 mm). In addition, T1-weighted structural images were acquired using a three-dimensional magnetization-prepared rapid-acquisition gradient echo (3D MPRAGE) sequence (TR = 1900 ms, TE = 2.52 ms, TI = 1100 ms, voxel size = 1 × 1 × 1 mm^3^, matrix size = 256 × 256). In the scanner, participants viewed the stimuli through the high-resolution stereo 3D glasses of the VisuaStim Digital MRI Compatible fMRI system.

### Facial and Bodily Expression Stimuli

**FIGURE 1:**
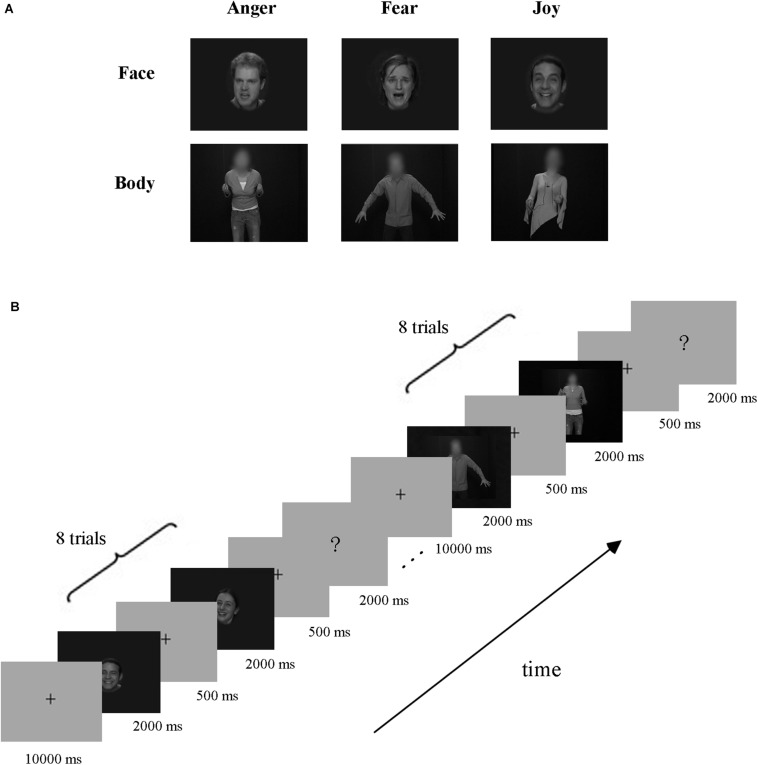
Exemplar stimuli and schematic representation of the experiment paradigm. **(A)** Exemplar facial and bodily expression stimuli. All emotion stimuli were taken from the GEMEP database. Videos of faces and bodies displaying three emotions (anger, fear, and joy) were used in the experiment. **(B)** Paradigm of the experiment design. A cross was presented for 10 s before each block, and then eight emotion stimuli appeared. Subsequently, the participants completed a button task to indicate their discrimination of the emotion category they had seen in the previous block.

Video clips of eight different individuals (four males and four females) displaying anger, fear, and joy (Grezes et al., 2007; de Gelder et al., 2012, 2015) were chosen from the Geneva Multimodal Emotion Portrayals (GEMEP) corpus (Banziger et al., 2012). Facial and bodily expression stimuli were created by cutting out and obscuring the irrelevant part of the whole-person videos using Gaussian blur masks (with Adobe Premiere Pro CC) (Kret et al., 2011), and the acquired face clips were magnified appropriately. All videos were edited to 2000 ms (25 frames/s) and were converted into grayscale using MATLAB (Furl et al., 2012, 2013, 2015; Kaiser et al., 2014; Soria Bauser and Suchan, 2015). Finally, video clips were resized to 720 × 576 pixels and presented on the center of the screen. All generated stimuli were validated well, with recognition from another group of participants before scanning. The exemplar stimuli are shown in Figure 1A.

### Experiment Paradigm

The experiment employed a block design, with three runs. There were three emotions (joy, anger, and fear) in our experiment, which were expressed by three stimulus types: facial expressions, bodily expressions, and whole-person expressions. Data from blocks of whole-person expressions were not analyzed in this study, which were included for the purpose of another study (Yang et al., 2018). The schematic representation of the experiment paradigm is shown in Figure 1B. Each run contained 18 blocks (3 emotions × 3 types × 2 repetitions), presented in a pseudo-random order to ensure that the same emotion or stimulus type did not appear consecutively (Axelrod and Yovel, 2012; Furl et al., 2013, 2015). At the beginning of each run, there was a 10 s fixation cross, followed by a stimulus block of eight trials, and then a 2 s button task. Successive stimulus blocks were separated by a fixation interval for 10 s. Each trial consisted of a 2000-ms stimulus video and an interstimulus interval (ISI) of 500 ms. During the button task, participants were instructed to press a button to indicate the emotion category they had seen in the previous block. Stimulus presentation was performed using E-Prime 2.0 Professional (Psychology Software Tools, Pittsburgh, PA, United States) and the behavioral responses were collected using the response pad in the scanner.

### Network Node Definitions

Regions of interests were defined according to the Brainnetome Atlas (Fan et al., 2016). The Brainnetome is generated using a connectivity-based parcellation framework, which provides fine-grained information on both anatomical and functional connections. We employed this atlas to define the network nodes in the FC analysis since it provided a stable starting point for the exploration of the complex relationships between structure, function and connectivity. There are 246 regions in this atlas, with 210 cortical and 36 subcortical subregions of the entire brain. Details about the label and the MNI coordinates of each node can be found at http://atlas.brainnetome.org/.

### Data Preprocessing

The fMRI data were first preprocessed using SPM8 software^1^. For each run, the first five volumes were discarded to allow for T1 equilibration effects (Wang et al., 2016; Liang et al., 2018; Zhang et al., 2018). The remaining functional images were corrected for slice acquisition time and head motion. Each participant’s structural image was co-registered with the functional images and was subsequently segmented into gray matter, white matter (WM), and cerebrospinal fluid (CSF). Then, the generated parameters by unified segmentation were used to normalize the functional images into the standard Montreal Neurological Institute (MNI) space with voxel sizes resampled into 3 × 3 × 3 mm. Finally, the functional data were spatially smoothed with a 4 mm full-width at half-maximum (FWHM) isotropic Gaussian kernel.

### Estimation of the FC Patterns for Facial and Bodily Expressions

The task-related whole-brain FC pattern estimation was carried out in MATLAB using the CONN toolbox^2^ (Whitfield-Gabrieli and Nieto-Castanon, 2012). CONN provides a common framework to perform a large suite of connectivity analyses for both resting and task fMRI data. For each subject, the normalized structural volume and the preprocessed functional images were submitted to CONN. A total of 246 network nodes were defined according to the Brainnetome Atlas. In FC analysis, it is critical to appropriately address noise in order to avoid possible confounding effects. CONN employs a component-based noise correction (CompCor) strategy (Behzadi et al., 2007), which can be particularly useful to reduce non-neural confounders in the context of FC analysis, increasing not only the validity, but also the sensitivity and specificity of the analysis. Before the FC calculation, standard preprocessing and denoising procedures using the default settings of the CONN toolbox were performed on the fMRI time series to further remove unwanted motion (Power et al., 2012) and physiological and other artificial effects from the BOLD signals. Confounding factors were regressed out by adding covariates of the six realignment parameters of head motion, the principal components of WM and CSF, and the first-order linear trend. The modeled task effects (box-car task design function convolved with the canonical hemodynamic response function) were also included as covariates to ensure that temporal correlations reflected FC and did not simply reflect task-related co-activations (Cole et al., 2019). Each of these defined confounding factors was then regressed out from the BOLD time series, and the resulting residual time series were temporally filtered using band-pass filter 0.01–0.1 Hz (Wang et al., 2016; Liang et al., 2018). The FC computation was conducted on the residual BOLD time series. After these preprocessing, the BOLD time series were divided into scans associated with each block presentation. All of the scans with nonzero effects in the resulting time series were concatenated for each condition (each emotion category in each stimulus type) and across all runs. Mean time series were obtained by averaging the time series of all voxels within each ROI and an ROI-to-ROI analysis was conducted to calculate the pairwise correlations between the mean time series of ROIs. Then, the correlation coefficients were Fisher *z* transformed, producing a connectivity map per emotion for each stimulus type in each participant, which were used as features in the later multivariate connectivity pattern classification analysis (fcMVPA).

### FcMVPA Classification Implementation

**FIGURE 2:**
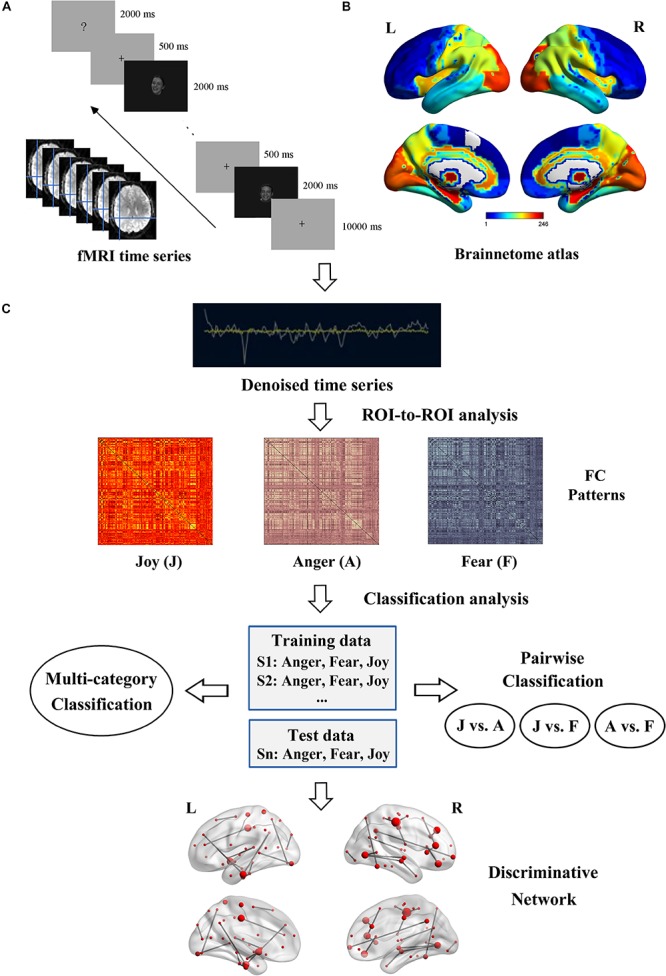
Flowchart of the experiment and data analysis procedure. **(A)** Experiment and fMRI data acquisition. **(B)** Brainnetome atlas for network nodes definition. **(C)** Framework overview of the fcMVPA. Estimation of the FC patterns was carried out using CONN toolbox. Before the FC computing, BOLD time series were denoising to further remove unwanted motion and physiological and other artifactual effects. Then, the whole-brain FC patterns for each emotion were constructed using ROI-to-ROI analysis. Emotion classification was performed in a leave-one-subject-out cross-validation scheme with an SVM classifier. Multi-category and pairwise emotion classifications for the facial and bodily expressions were conducted. Emotion-preferring networks were constructed based on the discriminative FCs.

Multivariate connectivity pattern classification was conducted on the estimated FC patterns to explore their roles in decoding of facial and bodily expressions. Figure 2 illustrates the flowchart of the experiment and analysis procedures, in which Figures 2A,B respectively show the fMRI data acquisition and the network nodes definition, and Figure 2C shows the overall framework of the fcMVPA. Due to the symmetry of the FC matrices, we extracted the lower triangle values to generate initial FC features. This procedure resulted in 30,135 [(246 × 245)/2] features in total. As there was evidence that showed that the interpretation of negative FCs remained controversial (Fox et al., 2009; Weissenbacher et al., 2009; Wang et al., 2016; Parente et al., 2018), in this study, we employed one-sample *t* tests to focus on the positive FCs and explored their roles in the decoding of facial and bodily expressions (Wang et al., 2016; Zhang et al., 2018). That is, for the training data, we conducted one-sample *t* test across participants for each of the 30,135 connections in each emotion category, and retained the FCs that had values significantly greater than zero [*p* values were corrected using false discovery rate (FDR) *q* = 0.01 for multiple comparisons]. Then, for each stimulus type (facial and bodily expressions), we pooled the positive FCs of the three emotions together to generate features for classification, which were significantly positive for at least one emotion category (Wang et al., 2016; Liang et al., 2018; Zhang et al., 2018). For the classification analysis, a linear support vector machine implemented in LIBSVM^3^ was employed as classifier, and the classification performance was evaluated with leave-one-subject-out cross-validation (LOOCV) scheme (Liu et al., 2015; Wang et al., 2016; Zhang et al., 2018). For each LOOCV fold, feature selection was only executed on the training set to avoid peeking. We conducted multi-category and pairwise emotion classifications for the facial and bodily expressions. The implementation of the multi-category classification employed a one-against-one voting strategy. In each LOOCV trial, the classifier was trained on all but one subject, and was tested on the remaining one. This procedure was repeated with 20 iterations; all subjects had been used as test data once, and the decoding performance was generated by averaging the accuracies of all iterations.

To evaluate the decoding performance, the statistical significance of the classification accuracy was assessed using permutation test (Liu et al., 2015; Cui et al., 2016; Wang et al., 2016; Fernandes et al., 2017). Permutation test is a non-parametric approach (Golland and Fischl, 2003), which is used to test the null hypothesis that the computed result is obtained by chance (Zhu et al., 2008). Thus, the same cross-validation procedure was carried out for 1000 random shuffles of class labels and the results were obtained across all permutations. If less than 5% of the accuracies from all permutations exceeded the actual accuracy (using correct labels), the result was considered to be significant (*p* < 0.05).

### Constructing Emotion-Preferring Networks for Facial and Bodily Expressions From Discriminative FCs

In this section, we identified the most contributive FCs in the emotion-discriminative networks for the facial and bodily expressions. We used the classification weights for each FC to reflect its contribution to the classification (Liu et al., 2015; Cui et al., 2016). Since feature selection was based on a slightly different sample subset in each LOOCV fold, consensus FCs that were selected on all folds were defined as the discriminative features. The discriminative weight for each feature was defined as the average of their absolute weights across all LOOCV folds. FCs with higher discriminative weights were considered to be more contributive to the emotion classification (Ecker et al., 2010; Dai et al., 2012; Cui et al., 2016). We then defined emotion-preferring network for each emotion category with FCs exhibiting reliable discriminative power when classifying a particular emotion with each of other two emotions.

## Results

### Behavioral Results

We collected the behavioral data for the recognition of facial and bodily expressions during the fMRI scanning. The average classification accuracy for facial expressions was 98.06% (SD = 3.73%) (Joy: mean = 100%, SD = 0; Anger: mean = 97.5%, SD = 6.11%; Fear: mean = 96.67%, SD = 6.84%), and that for bodily expressions was 97.22% (SD = 4.6%) (Joy: mean = 97.5%, SD = 6.11%; Anger: mean = 97.5%, SD = 6.11%; Fear: mean = 96.67%, SD = 6.84%). These results verified the validity of the stimuli in our experiment, where both facial and bodily expressions could be successfully classified at high accuracies. Further repeated-measures analysis of variance (ANOVA) for accuracies with Condition (Facial and Bodily) × Emotion (Joy, Anger, and Fear) revealed no significant effect for Condition [*F*(1,19) = 0.588, *p* = 0.453] or for Emotion [*F*(2,38) = 1.498, *p* = 0.237], and there was no significant interaction of Condition and Emotion [*F*(2,38) = 1, *p* = 0.357]. Details for the recognition accuracies and the corresponding reaction times can be found in Table 1.

**TABLE 1:** Behavioral accuracies and reaction times for facial and bodily expressions [mean% and standard deviation (SD)].

		**Classification**		**Reaction**	
		**accuracy (%)**		**time (ms)**	
		**Mean**	**SD**	**Mean**	**SD**
Facial expressions	Joy	100	0	713.74	163.39
	Anger	97.5	6.11	808.22	235.30
	Fear	96.67	6.84	809.54	234.73
	Total	98.06	3.73	777.17	200.75
Bodily expressions	Joy	97.5	6.11	669.36	160.87
	Anger	97.5	6.11	762.83	227.69
	Fear	96.67	6.84	825.16	220.20
	Total	97.22	4.60	752.45	188.85

### Emotion Classification Performance Based on fcMVPA

In this section, we conducted fcMVPA to explore the classification of facial and bodily expressions based on the constructed FC patterns. Network nodes were defined by the Brainnetome atlas and the fMRI time series were denoised using CONN to further remove unwanted motion (Van Dijk et al., 2012; Zeng et al., 2014) and physiological and other artificial effects from the BOLD signals. Table 2 summarizes the head motion parameters for different emotion categories. ROI-to-ROI analysis was performed to generate the connectivity map for each emotion in each stimulus type (facial and bodily expressions). In the main fcMVPA classification, we focused on the positive FCs (using one-sample *t* test with FDR *q* = 0.01) as features since the interpretation of negative FCs remained controversial and unclear (Fox et al., 2009; Weissenbacher et al., 2009; Wang et al., 2016; Parente et al., 2018). Table 3 shows the multi-category and pairwise classification results for the facial and bodily expressions based on the positive FCs. We also add analyses of an additional feature selection with *F* score (Liu et al., 2015), and these results are shown in Table 3 as *F* score results. Using the positive FCs, we found that both facial and bodily expressions could be successfully decoded for the multi-category emotion classification (chance level: 33.33%, *p* < 0.05, 1000 permutation tests), and for the pairwise emotion classification (chance level: 50%), all pairs of facial expressions (anger vs. fear, anger vs. joy, fear vs. joy) and two pairs of bodily expressions (anger vs. fear, fear vs. joy) could be significantly decoded (*p* < 0.05, 1000 permutation tests). In addition, we verified our analysis with two other parcellation schemes, the Harvard–Oxford atlas and the 200-region parcellations in Craddock et al. (2012), which were frequently used in previous fcMVPA studies (Wang et al., 2016; Liang et al., 2018; Zhang et al., 2018). These classification accuracies were generally similar to those from the Brainnetome atlas and were significantly higher than chance (decoding accuracies for the facial expressions were much higher than that for the bodily expressions), indicating the robustness of our results (Supplementary Table S1).

**TABLE 2:** Head motion parameters for different emotion categories (mean and SD).

		**Translation**			**Rotation**		
		***x* (mm)**	***y* (mm)**	***z* (mm)**	**Pitch (°)**	**Roll (°)**	**Yaw (°)**
Facial expressions	Joy	0.15 (0.12)	0.06 (0.03)	0.27 (0.18)	0.25 (0.14)	0.15 (0.09)	0.13 (0.10)
	Anger	0.13 (0.09)	0.06 (0.02)	0.23 (0.16)	0.24 (0.16)	0.13 (0.07)	0.11 (0.07)
	Fear	0.16 (0.11)	0.07 (0.03)	0.29 (0.21)	0.29 (0.19)	0.16 (0.09)	0.14 (0.11)
Bodily expressions	Joy	0.16 (0.11)	0.06 (0.03)	0.27 (0.18)	0.26 (0.14)	0.15 (0.08)	0.14 (0.10)
	Anger	0.16 (0.12)	0.07 (0.03)	0.26 (0.15)	0.25 (0.14)	0.15 (0.08)	0.14 (0.10)
	Fear	0.14 (0.10)	0.06 (0.03)	0.23 (0.17)	0.24 (0.15)	0.13 (0.07)	0.12 (0.09)

**TABLE 3:** Accuracies of decoding facial and bodily expressions using fcMVPA.

	**Facial expressions**		**Bodily expressions**	
	**Positive FCs**	***F* score**	**Positive FCs**	***F* score**
**Multi-category classification (Chance level: 33.33%)**				
	56.67%^*^	53.33%^*^	43.33%^*^	46.67%^*^
**Category pairwise classification (Chance level: 50%)**				
Anger–Fear	70%^∗^	72.5%^*^	62.5%^*^	72.5%^*^
Anger–Joy	60%^∗^	60%^∗^	52.5%	70%^∗^
Fear–Joy	77.5%^*^	80%^∗^	70%^∗^	75%^∗^

*^∗^Significant results at *p* < 0.05 over 1000 permutation tests.*

**FIGURE 3:**
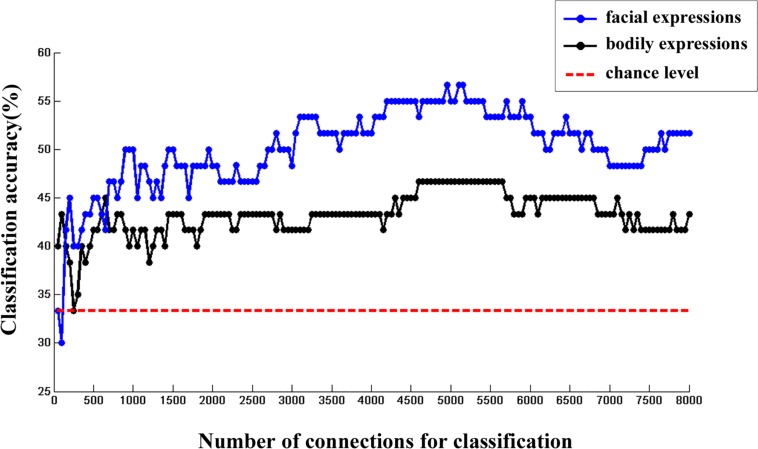
The changes of multi-category classification accuracies for facial and bodily expressions when different number of FC features were used.

Moreover, we calculated the multi-category classification accuracy as a function of the number of FC features used in the classification procedure. In this step, FC features were ranked according to their *p* values of one-sample *t* test in ascending order. Results are shown in Figure 3. We found that both facial and bodily expressions could be consistently successful in decoding based on the large-scale FC patterns, and the decoding accuracies were higher for the facial than for the bodily expressions.

### Discriminative Networks for Facial and Bodily Expressions

**FIGURE 4:**
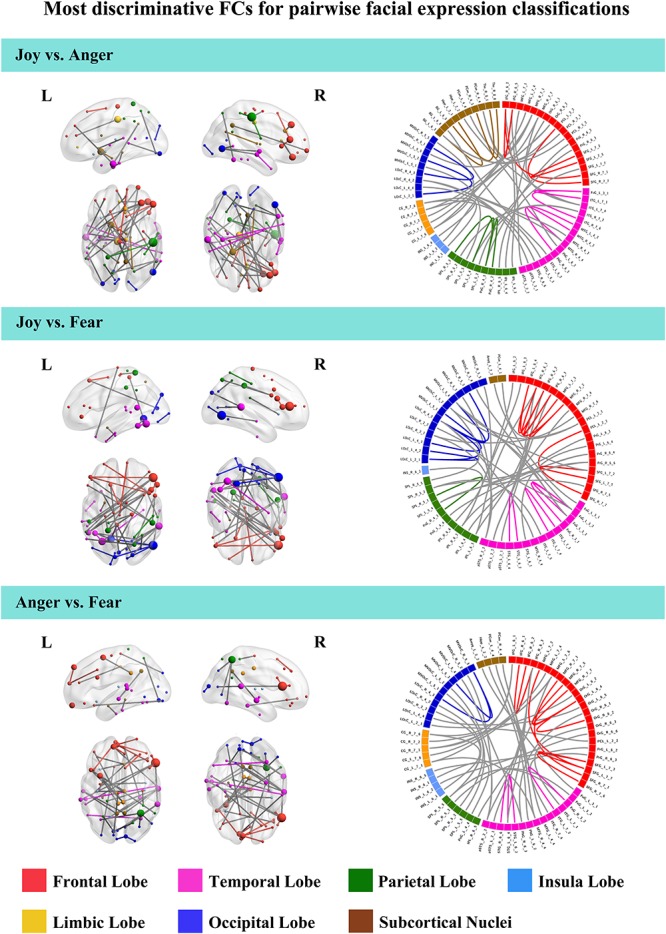
Most discriminative FCs for pairwise facial expression classifications. Results are mapped onto the cortical surfaces using BrainNet Viewer. The coordinates of each node are according to the Brainnetome atlas, and the brain regions are scaled by the number of their connections. The connectogram is created using Circos. Different colors are used to indicate different modules (the frontal, temporal, parietal, insula, limbic and occipital lobes as well as the subcortical nuclei) according to the Brainnetome atlas. Lines of the intra-module connections are represented by the same color as the located module, while the inter-module connections are represented by gray lines.

**FIGURE 5:**
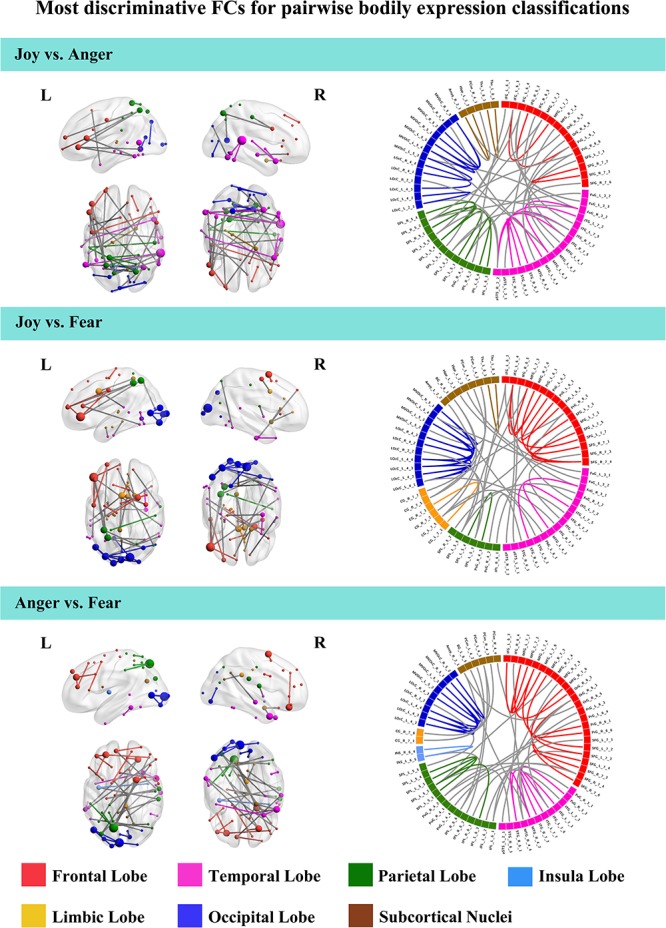
Most discriminative FCs for pairwise bodily expression classifications. Results are mapped onto the cortical surfaces using BrainNet Viewer. The coordinates of each node are according to the Brainnetome atlas and the brain regions are scaled by the number of their connections. The connectogram is created using Circos. Different colors are used to indicate different modules (the frontal, temporal, parietal, insula, limbic, and occipital lobes as well as the subcortical nuclei) according to the Brainnetome atlas. Lines of the intra-module connections are represented by the same color as the located module, while the inter-module connections are represented by gray lines.

To further understand the emotion-discriminative networks for facial and bodily expressions, we identified the most contributive FCs based on the classifier weights. Consensus FCs were firstly selected on all folds of LOOCV, and the discriminative weight for each feature was defined as the average of their absolute weights across all folds of classification (Ecker et al., 2010; Dai et al., 2012; Cui et al., 2016). FCs with higher discriminative weights were considered to be more contributive to the emotion classification. Figures 4, 5 show the top 50 most contributive FCs (mapped onto the cortical surfaces using BrainNet Viewer, Xia et al., 2013, and the connectogram is created using Circos)^4^ based on the discriminative weights for the pairwise emotion classifications (joy vs. anger, joy vs. fear, anger vs. fear). Different colors are used to indicate different modules (the frontal, temporal, parietal, insula, limbic, and occipital lobes as well as the subcortical nuclei) according to the Brainnetome atlas. Lines of the intra-module connections are represented by the same color as the located module, while the inter-module connections are represented by gray lines. With insight into these emotion-discriminative networks, we found the involvement of widespread brain regions in both hemispheres, ranging from primary visual regions to higher-level cognitive regions. Furthermore, we identified emotion-preferring network for each emotion category based on these contributive FCs, constituting with FCs that exhibited reliable discriminative power when classifying a particular emotion with each of the other two emotions (details of the discriminative FCs for each emotion are summarized in Table 4). We found that, for both facial and bodily expressions, fear engaged more discriminative FCs than anger and joy. Moreover, we compared the emotion-preferring networks between the facial and bodily expressions. We found that, for a particular emotion, discriminative FCs for facial and bodily expressions were distinct, suggesting that emotions perceiving from different body cues are processed rely upon different networks.

**TABLE 4:** Discriminative FCs for the facial and bodily expressions.

	**Node A**				**Node B**			
	***x***	***y***	***z***	**Label**	***x***	***y***	***z***	**Label**
**Facial expressions**								
**Joy**								
1	48	–70	–1	R Lateral Occipital Cortex	58	–16	–10	R Middle Temporal Gyrus
2	8	58	13	R Superior Frontal Gyrus	–4	–23	61	L Paracentral Lobule
3	48	35	13	R Inferior Frontal Gyrus	–51	–33	42	L Inferior Parietal Lobule
4	–6	–5	58	L Superior Frontal Gyrus	–18	24	53	L Superior Frontal Gyrus
**Anger**								
1	–7	–23	41	L Cingulate Gyrus	–41	41	16	L Middle Frontal Gyrus
2	6	–20	40	R Cingulate Gyrus	–36	–20	10	L Insular Gyrus
3	–11	–82	–11	L MedioVentral Occipital Cortex	–27	–59	54	L Superior Parietal Lobule
4	–28	–30	–10	L Hippocampus	–25	–25	–26	L Parahippocampal Gyrus
**Fear**								
1	–27	–4	–20	L Amygdala	–33	–16	–32	L Fusiform Gyrus
2	29	–75	36	R Lateral Occipital Cortex	31	–54	53	R Superior Parietal Lobule
3	57	–40	12	R Posterior Superior Temporal Sulcus	7	–76	11	R MedioVentral Occipital Cortex
4	57	–40	12	R Posterior Superior Temporal Sulcus	–5	–81	10	L MedioVentral Occipital Cortex
5	–62	–33	7	L Superior Temporal Gyrus	–52	–32	12	L Superior Temporal Gyrus
6	54	24	12	R Inferior Frontal Gyrus	48	35	13	R Inferior Frontal Gyrus
7	54	24	12	R Inferior Frontal Gyrus	45	16	25	R Inferior Frontal Gyrus
**Bodily expressions**								
**Joy**								
1	–54	–40	4	L Posterior Superior Temporal Sulcus	42	22	3	R Inferior Frontal Gyrus
2	–33	–47	50	L Superior Parietal Lobule	–28	56	12	L Middle Frontal Gyrus
3	–27	–59	54	L Superior Parietal Lobule	–49	36	–3	L Inferior Frontal Gyrus
4	–16	–24	6	L Thalamus	–18	–23	4	L Thalamus
**Anger**								
1	19	–2	–19	R Amygdala	9	20	–19	R Orbital Gyrus
2	7	–76	11	R MedioVentral Occipital Cortex	10	–85	–9	R MedioVentral Occipital Cortex
3	–15	–71	52	L Superior Parietal Lobule	42	44	14	R Middle Frontal Gyrus
4	–22	–47	65	L Superior Parietal Lobule	–16	–60	63	L Superior Parietal Lobule
**Fear**								
1	34	8	54	R Middle Frontal Gyrus	20	4	64	R Superior Frontal Gyrus
2	51	–4	–1	R Superior Temporal Gyrus	56	–10	15	R Postcentral Gyrus
3	–18	–99	2	L Lateral Occipital Cortex	–6	–94	1	L MedioVentral Occipital Cortex
4	–18	–99	2	L Lateral Occipital Cortex	–46	–74	3	L Lateral Occipital Cortex
5	22	–97	4	R Lateral Occipital Cortex	–6	–94	1	L MedioVentral Occipital Cortex
6	29	–75	36	R Lateral Occipital Cortex	–27	–59	54	L Superior Parietal Lobule
7	–30	–88	–12	L Lateral Occipital Cortex	–46	–74	3	L Lateral Occipital Cortex
8	–30	–88	–12	L Lateral Occipital Cortex	–6	–94	1	L MedioVentral Occipital Cortex

*The label and MNI coordinates of each node are according to the Brainnetome Atlas.*

## Discussion

In the present study, we explored network representation mechanisms for facial and bodily expressions based on the FC analysis. We employed a continuous multi-category emotion task paradigm wherein participants viewed emotions (joy, anger, and fear) from facial and bodily expressions. We constructed the FC patterns for each emotion in each stimulus type and conducted multivariate connectivity pattern classification analysis (fcMVPA). Results showed that the FC patterns made successful predictions of emotion categories for both facial and bodily expressions, and the decoding accuracies were higher for the facial than for the bodily expressions. Further discriminative FC analysis showed the involvement of a wide range of brain areas in the emotion processing, and the emotion-preferring networks for facial and bodily expressions were different.

### Successful Decoding of Facial and Bodily Expressions Based on the Large-Scale FC Patterns

Adopting FC-based MVPA, we showed that emotions perceiving from facial and bodily expressions can be successfully decoded from the large-scale FC patterns.

Regarding the exploration of the neural basis for the emotion perception, most of the previous neuroimaging studies were dominated by using activation-based univariate analysis to identify brain regions showing significant responses to facial or bodily expressions (Kanwisher and Yovel, 2006; de Gelder et al., 2010; Kret et al., 2011; Pitcher, 2014; Henriksson et al., 2015; Downing and Peelen, 2016; Tippett et al., 2018). Although some recent studies employed machine learning algorithms into fMRI analysis, they mainly focused on the activation-based decoding of facial emotions in several predefined ROIs (Said et al., 2010; Harry et al., 2013; Wegrzyn et al., 2015; Liang et al., 2017). Due to the expected existence of interactions between distinct cortical regions, FC analysis has recently attracted more and more interest. A growing body of evidence suggests that distinct cortical regions are intrinsically interconnected during the processing of high-level cognition (Cole et al., 2013; Wang et al., 2016; Zhang et al., 2018). One of our recent studies employed FC-based analysis and showed successful decoding of facial expressions based on the large-scale FC patterns (Liang et al., 2018). To date, however, compared with facial expressions, bodily expressions have received relatively little attention, and no fMRI studies have adequately addressed the potential role of the FC patterns in the decoding of bodily expressions. In the present study, using whole-brain FC analysis and fcMVPA classification, we found that in addition to the facial expressions, bodily expressions could also be successfully decoded from the large-scale FC patterns. These results add to the recently increasing number of studies suggesting that significant amount of information may also be represented in the FC patterns, which can be successfully applied to distinguish social anxiety disorder and major depression patients from the healthy controls (Zeng et al., 2012; Liu et al., 2015), and differentiate among various object categories (Wang et al., 2016), tasks (Cole et al., 2013), mental states (Dosenbach et al., 2010; Pantazatos et al., 2012; Shirer et al., 2012), and sound categories (Zhang et al., 2018). Moreover, our results not only are in line with previous findings on facial expressions but also further suggest the potential contribution of the large-scale FC patterns in the processing of bodily expressions.

Taken together, our results highlight the potential role of the FC patterns in the neural processing of emotions, suggesting that large-scale FC patterns may contain rich emotional information to accurately decode both facial and bodily expressions. Our study provides new evidence for the distributed neural representations of emotions in the large-scale FC patterns and further support that general interactions between distributed brain regions may effectively contribute to the decoding of human emotions.

### Network Representations for Facial and Bodily Expressions

In this study, we identified the most contributive FCs in emotion discrimination based on the classifier weights. Figures 4, 5 show the top 50 most discriminative FCs (mapped onto the cortical surfaces using BrainNet Viewer, Xia et al., 2013, and the connectogram is created using Circos, see text footnote 4) for the pairwise emotion classifications (joy vs. anger, joy vs. fear, anger vs. fear). Different colors are used to indicate different brain modules (the frontal, temporal, parietal, insula, limbic, and occipital lobes as well as the subcortical nuclei) according to the Brainnetome atlas. Lines of the intra-module connections are represented by the same color as the located module, while the inter-module connections are represented by gray lines. We found that these emotion-discriminative networks were widely distributed in both hemispheres, containing FCs among widespread brain regions in occipital, parietal, temporal, and frontal lobes, ranging from primary visual areas to higher-level cognitive areas. Particularly, these networks included classical face- and body-selective areas, such as the FG and the posterior superior temporal sulcus (pSTS). Additionally, regions that were not classically considered sensitive by traditional activation-based measures, such as the postcentral gyrus and the middle frontal gyrus, were also included in the discriminative networks. To some extent, these results were compatible with recent fcMVPA studies on decoding of various object categories, sounds, and facial expressions, suggesting the potential effects of the activation-defined neutral areas on high-level cognition (Wang et al., 2016; Liang et al., 2018; Zhang et al., 2018). Together, our results from the discriminative network analysis indicate how large-scale FC patterns reconfigure in the processing of facial and bodily expressions and further corroborate the distributed neural representation for the emotion recognition.

Furthermore, we constructed an emotion-preferring network for each emotion category, composed of FCs that significantly contributed to the classifications between a particular emotion and the other two emotion categories (Table 4). With insight into these emotion-preferring networks, we found that for the facial expressions, joy evoked FCs across the occipital, the frontal, the temporal, and the parietal lobes; fear evoked more FCs than joy and anger, which is mainly across the occipital, the temporal, the frontal, and the parietal lobes as well as the subcortical nuclei; and anger evoked FCs across all seven modules. Our results were compatible with previous studies on facial expression perception, which demonstrated the involvement of anatomical regions, such as the visual areas, the FG, the STS, the amygdala, the insula, the middle temporal gyrus, and the inferior frontal areas, in the processing, analyzing, and evaluating of the emotional facial stimuli (Trantmann et al., 2009; Kret et al., 2011; Harry et al., 2013; Furl et al., 2015; Henriksson et al., 2015; Wegrzyn et al., 2015; Liang et al., 2017). Moreover, results of our emotion-preferring networks may provide new evidence to indicate the potential preference of a specific region in the processing of particular emotions; for instance, in our study, amygdala was involved in the discriminative network for fear, which was consistent with previous findings that showed that amygdala could enhance the encoding of fearful facial expressions using dynamic causal modeling analysis (Furl et al., 2013, 2015).

For the emotion-preferring networks of bodily expressions, however, we found that the networks for each emotion were different from that for the facial expressions. When perceiving emotions from bodily stimuli, joy mainly evoked FCs across the temporal, the parietal, and the frontal lobes as well as the subcortical nuclei; anger evoked FCs across the subcortical nuclei, the occipital, the parietal, and the frontal lobes; and fear mainly evoked FCs across the frontal, the temporal, the occipital, and the parietal lobes. For fear, the bilateral lateral occipital cortex served as the hub region (most densely connected region). Previous studies using activation-based analysis have found the preferential activations in the STS, the superior parietal lobule, the superior temporal gyrus, and the thalamus for the bodily expression perception (Kret et al., 2011; Yang et al., 2018). Our results were consistent with these previous findings, and may further our understanding of the neural basis for decoding of bodily expressions. Moreover, for a particular emotion, discriminative FCs for facial and bodily expressions were distinct, suggesting that the human brain employs separate network representations for facial and bodily expressions of the same emotions. To sum, our results provide new evidence for the network representations of emotions, and suggest that emotions perceiving from different body cues may be processed rely upon different networks.

The present study employed a similar sample size as those reported in previous fMRI studies on facial emotion perception and fcMVPA-based decoding analyses (Furl et al., 2013; Wegrzyn et al., 2015; Wang et al., 2016; Zhang et al., 2018). The inclusion of additional samples could further improve the statistical power and boost the accuracy. Moreover, a larger number of participants can better prove the effectiveness of our findings. Thus, it is important to confirm our findings with a larger sample size in the future study. Additionally, emotions can also be perceived from sounds and other clues. Future studies on other perceptual cues would be meaningful to help to further understand the neural basis of emotion processing more fully and deeply.

## Conclusion

Taken together, using fcMVPA-based classification analyses, we show that rich emotional information is represented in the large-scale FC patterns, which can accurately decode not only facial but also bodily expressions. These findings further corroborate the importance of the FC patterns in emotion perception. In addition, we show that the emotion-discriminative networks are widely distributed in both hemispheres, suggesting the interactive nature of distributed brain areas underlying the neural representations of emotions. Furthermore, our results provide new evidence for the network representations of facial and bodily expressions and suggest that emotions perceiving from different body cues may be processed rely upon different networks. This study further extends previous fcMVPA studies and may be helpful to improve the understanding of the potential mechanisms that enable the human brain to efficiently recognize emotions from body language in daily lives.

## Data Availability Statement

The datasets generated for this study are available on reasonable request to the corresponding author.

## Ethics Statement

The studies involving human participants were reviewed and approved by the Research Ethics Committee of Yantai Affiliated Hospital of Binzhou Medical University. The patients/participants provided their written informed consent to participate in this study.

## Author Contributions

BL designed the study. YL and XL performed the experiments. YL analyzed the results and wrote the manuscript. YL and JJ contributed to the manuscript revision. All authors have approved the final manuscript.

## Conflict of Interest

The authors declare that the research was conducted in the absence of any commercial or financial relationships that could be construed as a potential conflict of interest.

## Footnotes

1 http://www.fil.ion.ucl.ac.uk/spm/software/spm8/

2 http://www.nitrc.org/projects/conn

3 https://www.csie.ntu.edu.tw/~cjlin/libsvm/

4 http://circos.ca/
