# Supplementary material for: Network Representations of Facial and Bodily Expressions: Evidence From Multivariate Connectivity Pattern Classification
Source: Front Neurosci. 2019 Oct 29;13:1111. doi: 10.3389/fnins.2019.01111 (PMC6828617; doi:10.3389/fnins.2019.01111)
Supplement: Supplementary file 1 [file Table_1.docx]

**Supplemental Table 1. Decoding accuracies for facial and bodily expressions from positive FCs of other brain atlases**

|  | Facial expressions | | |  | Bodily expressions | | |
| --- | --- | --- | --- | --- | --- | --- | --- |
|  | HOA |  | CC200 |  | HOA |  | CC200 |
| Multi-category classification (Chance level: 33.33%) | | | | | | | |
|  | 55% |  | 65% |  | 45% |  | 46.67% |
| Category pairwise classification (Chance level: 50%) | | | | | | | |
| Anger-Fear | 75% |  | 82.5% |  | 67.5% |  | 65% |
| Anger-Joy | 67.5% |  | 70% |  | 55% |  | 72.5% |
| Fear-Joy | 70% |  | 87.5% |  | 65% |  | 70% |

HOA: Harvard-Oxford cortical and subcortical structural atlas; CC200: Craddock 200-region parcellations.
